# Effect of Antihypertensive Losartan on Ca^2+^ Mobilization in the Aorta of Middle-Aged Spontaneously Hypertensive Female Rats

**DOI:** 10.3390/jcdd12110441

**Published:** 2025-11-07

**Authors:** Swasti Rastogi, Jessica Liaw, Yingnan Zhai, Tatiana Karpova, Linxia Gu, Kenia Nunes

**Affiliations:** 1 Laboratory of Vascular Biology, Department of Biomedical Engineering and Science, Florida Institute of Technology, Melbourne, FL 32901, USA; srastogi2022@my.fit.edu (S.R.);; 2 Laboratory of Biomechanics, Department of Biomedical Engineering and Science, Florida Institute of Technology, Melbourne, FL 32901, USAgul@fit.edu (L.G.)

**Keywords:** hypertension, vascular dysfunction, Ca^2+^ mishandling, middle-aged female, losartan, aortic stiffness

## Abstract

Hypertension, a leading factor for cardiovascular diseases (CVD), is a particularly heavy burden in women during middle age, when cardioprotective hormones begin to decline. The abnormal handling of calcium (Ca^2+^) in vascular smooth muscle cells (VSMCs) leads to increased vasoconstriction, remodeling, and altered arterial compliance during hypertension. The Spontaneously Hypertensive Rats (SHR) is a model of essential hypertension, and middle-aged females with hypertension represent a stage of disease where vascular dysfunction is prominent but understudied. Losartan, a widely prescribed angiotensin II (AngII) receptor (AT1R) blocker, exerts antihypertensive effects by affecting Ang II/Ca^2+^ signaling. However, whether it corrects the Ca^2+^ mishandling in the aorta of middle-aged female SHR has not been established. In this study, the thoracic aorta from 36-week-old female SHRs treated with losartan was assessed for Ca^2+^ mishandling using myography and biochemical assays. Meanwhile, biomechanical properties and stiffness were evaluated using Pulse Wave Velocity (PWV), Atomic Force Microscopy (AFM), and assessments of collagen and elastin contents. Compared with normotensive controls, SHR demonstrated disrupted Ca^2+^ handling, increased stiffness, and Extracellular Matrix (ECM) remodeling in middle-aged females. Treatment with losartan abrogated Ca^2+^ mishandling influx and efflux in the VSMC, decreased stiffness, and restored the aortic structural changes. These findings demonstrate that losartan abolishes Ca^2+^ mishandling and highlight a mechanistic role of AT1R blockade in restoring vascular function in the aorta of middle-aged females during hypertension.

## 1. Introduction

Hypertension is a major risk factor for CVD and remains a leading cause of morbidity and mortality worldwide [1]. It has been termed a silent killer because of its asymptomatic progression and strong association with organ damage [2,3]. Although hypertension has been studied widely, underlying mechanisms remain unclear, particularly with respect to age and sex. Sex differences in blood pressure have been reported, with women exhibiting a sharp increase in blood pressure parameters beginning in their 30s. [4]. This trend is particularly evident during the transition to menopause with increasing age, significantly impacting vascular function with an increased risk of hypertension in middle-aged individuals [5]. Despite these variables, females are still underrepresented in hypertension research, leaving a gap in understanding the mechanisms associated with vascular dysfunction during middle age.

One critical component implicated in the pathophysiology of hypertension-associated vascular dysfunction is disrupted Ca^2+^ signaling [6]. As a primary secondary messenger molecule, calcium’s cytosolic concentration directly determines the degree of contraction in all muscle types, including VSMCs [7]. Upon adrenergic stimulation, VSMCs elicit a biphasic contraction curve: an initial rapid phasic contraction facilitated by Ca^2+^ release from the sarcoplasmic reticulum (SR) into the cytosol (Ca^2+^ efflux), followed by a slow, sustained tonic phase mediated by Ca^2+^ release from voltage-dependent and -independent channels (Ca^2+^ influx) [8]. An illustration of the biphasic contraction curve is shown below in the results section. These components drive proper vascular reactivity and tone. Thus, dysregulation of either phase contributes to the development of vascular dysfunction.

In hypertension, this highly regulated process is disrupted. Calcium surplus alters basal vascular tone, impairs relaxation, decreases compliance, and can indirectly induce changes in ECM composition [9]. Aging further exacerbates these Ca^2+^-handling abnormalities, thereby worsening vascular dysfunction in the context of hypertension [10]. Therefore, impaired Ca^2+^ handling is central to understanding hypertension-related vascular dysfunction, as it can shed light, unraveling sex- and age-specific mechanisms associated with disease progression. Indeed, sex differences in Ca^2+^ handling have been reported in the SHR model, with females displaying reduced Ca^2+^ influx compared to males [11,12]. However, whether these differences persist into middle age, with more pronounced vascular dysfunction, remains unclear.

Losartan, an effective antihypertensive treatment that prevents Ang II-mediated vasoconstriction, exhibits sex-specific effects in hypertension. Studies have shown that female hypertensive rats respond more effectively to losartan than males [13]. Additionally, a post hoc analysis of the LIFE study reported that losartan was more effective than atenolol, a beta blocker, and reduced CVD events to a greater extent in hypertensive women compared to men, thereby improving the cardiovascular outcome [14]. AngII acts via AT1 receptor and elevates Ca^2+^ mobilization in VSMCs; disrupted Ca^2+^ handling is a central component of hypertensive vasculature [15,16]. Therefore, it is essential to further elucidate whether losartan affects Ca^2+^ mobilization in females with increasing age and disease progression. To date, no studies have explored this gap. Here, we investigate the impact of losartan on Ca^2+^ mobilization and arterial biomechanics by assessing changes in the aorta of 36-week (middle-aged) female SHRs at macro-, micro-, and nano-levels. Our results contribute to a better understanding of sex-specific vascular responses in the aorta of hypertensive females, thereby improving cardiovascular-related outcomes.

## 2. Materials and Methods

### 2.1. Animals

All animal procedures were followed as per the Guide for the Care and Use of Laboratory Animals from the National Institutes of Health. They were reviewed and approved by the Florida Institute of Technology Institutional Animal Care and Use Committees under the protocol (No. #2024-05, approval date 20 December 2024). All procedures were overseen by a qualified veterinarian, in accordance with ARRIVE guidelines [17]. Also, steps were taken to reduce pain and distress by adhering to the 3Rs (Replacement, Reduction, and Refinement). Middle-aged (36-week-old, n = 32) Female Wistar (WIS) rats served as the normotensive control, and SHR served as the hypertensive group, both acquired from Charles River and acclimated for 48 h before any procedures were performed. Animals were kept in a controlled environment with a 12 h light exposure cycle and free access to food and water. Body weight and blood pressure, including systolic blood pressure (SBP) and diastolic blood pressure (DBP), were measured in all animals throughout the study using the non-invasive tail-cuff method with the High-Throughput CODA System (Kent Scientific Corporation, Torrington, CT, USA). The animals were trained for at least 1 week before blood pressure was recorded with minimal restraint. Animals with systolic pressure exceeding 130 mmHg were considered to have elevated BP in accordance with AHA and ESC guidelines [18,19]. All measurements were performed by the same individual, and Mean Arterial Pressure (MAP) was calculated using the equation:

DBP + 1/3(SBP − DBP).

### 2.2. Losartan Treatment

The animals (n = 8 per group) were randomly assigned to one of the four groups at the age of 20 weeks: (1) WIS with vehicle, (2) WIS treated with losartan, (3) SHR with vehicle, and (4) SHR treated with losartan. Losartan (Sigma Aldrich, St Louis, MO, USA, No. SML3317) was administered via drinking water at a dosage of 10 mg/kg every day for a period of 4 months. The dose of losartan was selected based on previous studies conducted that showed its efficacy in reducing blood pressure and cardioprotective effects in SHRs [20,21,22]. Following the treatment period, the animals were anesthetized and euthanized. Four animals from each group were used for each experimental setup. Additionally, we kept one animal per cage to monitor the animals and ensure the correct dosage of treatment was administered.

### 2.3. Sample Preparation

The thoracic aorta was carefully excised, cleaned, and immediately snap-frozen in liquid nitrogen for later analysis of ECM-components such as elastin and collagen.

### 2.4. Functional Studies

The aorta was harvested from these animals and cut into 2 mm rings. The rings were then mounted in a multi-wire myograph system, DMT620M (Danish Myo Technology, Aarhus, Denmark), in a chamber filled with Physiological Salt Solution (PSS) at a resting tension of 30 mN and supplied with carbogen (95% CO_2_ and 5% O_2_) at a controlled temperature of 37 °C. During the 1 h stabilization period, the PSS was replaced every 15 min, and resting tension was adjusted accordingly. Subsequently, the rings were exposed to a 120 mM KCl solution to test their viability. Rings are considered viable if the generated force is greater than half of the preload tension. Next, the rings were rinsed and allowed to return to their baseline tension. They were then processed as described below.

#### 2.4.1. Concentration–Response Curve

Endothelium-intact aortic rings were used to generate a dose–response curve to phenylephrine (PE, 1 nmol/L to 100 μmol/L), as PE is a widely used α-1 adrenergic receptor agonist that induces vasoconstriction in isolated aortas.

#### 2.4.2. Time–Force Curves

The rings from the aorta were stimulated with a single dose of Phenylephrine (10 μmol/L), and the force generated was evaluated for 10 min. The biphasic contraction components: Phasic (fast) and Tonic (slow) contraction were determined. The phasic contraction component was calculated as the difference between the peak force during the rapid contraction phase and the initial force at time zero. The tonic component was determined by deducting the initial force at time zero from the force measured at the end of 10 min.

#### 2.4.3. Ca^2+^ Protocol

To evaluate Ca^2+^ mobilization, we applied a protocol in which extracellular Ca^2+^ was first removed and then reintroduced in the PSS. The aortic rings with intact endothelium were processed as described above until the viability test. The PSS was then replaced with a Ca^2+^-free solution, prepared by adjusting the PSS by adding 1 mmol/L EGTA and omitting the 1.56 mmol/L of CaCl_2_·H_2_O. Following 3 min, a single dose of PE (10 μmol/L) was applied to stimulate the samples. The force generated in response to phenylephrine was recorded for 10 min. Subsequently, the initial Ca^2+^ concentration was restored to the solution, and the force generated in response to Ca^2+^ influx was evaluated for 10 min. The maximum contraction for both phasic and tonic phases, reflective of Ca^2+^ release from sarcoplasmic reticulum (Ca^2+^ efflux) and Ca^2+^ entry through plasma membrane channel (Ca^2+^ Influx), was quantified. The Emax of PE, depicting Ca^2+^ efflux, was calculated by subtracting the force at time zero (when Ca^2+^ free PSS was added) from the peak force attained after addition of PE. For the Emax of Ca^2+^, the force measured at the end of 10 min (post addition of PE) was deducted from the peak force attained after the extracellular Ca^2+^ was restored in the myograph chamber. This protocol indirectly evaluates Ca^2+^ dynamics as a function of the change in force development.

### 2.5. Ca^2+^ Assay Kit

In a separate batch of experiments, aortic rings (2 mm long) were placed in a myograph chamber with PSS. To induce contraction, rings were then challenged with a single dose of PE (10 μmol/L) for 10 min and immediately snap-frozen in liquid nitrogen to preserve the tissue structure and halt the biochemical processes. Intracellular free Ca^2+^ levels were determined using the colorimetric Calcium Assay Kit (Abcam, Waltham, MA, USA, ab102505) according to the manufacturer’s protocol and as previously described in the literature [23]. Subsequently, the frozen tissues were pulverized in lysis buffer, homogenized, and centrifuged. The supernatant was collected and added to a 96-well plate in duplicates alongside standards following the instructions on the kit. Optical density (OD) was recorded at 575 nm on a plate reader (Molecular Devices, San Jose, CA, USA, SpectraMax i3). This assay provides an overview of free calcium levels in the vascular tissue during contraction.

### 2.6. Echocardiography

Echocardiography was carried out using a Vevo 3100 ultrasound system (FUJIFILM VisualSonics, Toronto, ON, Canada) equipped with an MX250 transducer (FUJIFILM VisualSonics, Toronto, ON, Canada). The animals were anesthetized with isoflurane 1–3% vol/vol in an induction chamber for 2–3 min until loss of righting reflex. They were then dorsally placed on a warm platform to stabilize their body temperature, with their nose in a nosecone, delivering 1.5–2% isoflurane throughout the procedure. The eyes were coated with a lubricant to prevent dryness, and their paws were taped down on the four electrocardiogram electrodes embedded in the platform to record heart rate, beats per minute, and respiratory rate. A depilatory cream was applied to the imaging site to remove the fur for 2–3 min. The imaging area was cleaned, and ultrasound gel was applied. The transducer was lowered until it touched down on the gel and placed at a 90° angle to obtain a long-axis (longitudinal) view. Imaging was performed after the losartan treatment to assess hemodynamics and aortic stiffness.

Aortic diameter was measured in B-mode while RI, PI, and PWV were derived from Doppler mode and analyzed with Vevo Lab Software 5.8.1 (FUJIFILM VisualSonics, Toronto, ON, Canada). For PWV, the transit time method was applied, as described previously [24,25,26]. Two points along the aorta were selected, and at each point, the time interval between the R-wave onset on the ECG and the foot of the Doppler waveform was measured. The transit time (Δt) was calculated as the difference between these two time intervals, and d is the distance between those two points. PWV was calculated as:

$$PWV=\frac{d\;(m)}{\Delta t\;(s)}$$

### 2.7. Atomic Force Microscopy

Atomic force microscopy (AFM) was performed using a JPK NanoWizard^®^ 4 system (Bruker, Billerica, MA, USA) equipped with Bruker Scanasyst-Fluid probes (tip radius: 20 nm, spring constant: 0.7 N/m, resonance frequency: 150 kHz), operated in QI mode, and analyzed using the manufacturer’s software version 8.0.192 (Bruker, Billerica, MA, USA). Aortic cryosections were thawed at room temperature for 5 min and followed by immersion in 1× PBS for 30 min to remove the OCT compound. AFM indentation tests were performed in 1× PBS. For each animal, 3 cryosections were obtained, and 3 regions were randomly selected within the medial layer of each section. Each region was tested in a 10 × 10 µm^2^ area and an 8 × 8-pixel grid, yielding 64 indentation points per test region, and 576 indentation points per group. Young’s modulus maps were then generated from these measurements.

For each indentation point, a corresponding force–distance curve was fitted with the Sneddon model to calculate Young’s modulus:

$$F=\frac{2}{\pi }\frac{E}{\left(1-\nu^{2}\right)}\tan\left(\alpha \right)\delta^{2}$$

where F is the measured indentation force, ẟ is the measured indentation depth, ν is Poisson’s ratio (0.49), α is the half-angle of the AFM probe tip (18°), and E is Young’s modulus, which was determined by fitting the indentation portion of the force–distance curve from the initial contact point to the force setpoint.

### 2.8. Paraffin Embedding

Thoracic aorta rings of 2 mm were dehydrated in 4% paraformaldehyde and dehydrated in graded ethanol (50% and 70%). Tissues were processed in histology cassettes using Leica TP1020 Automatic Benchtop Tissue Processor (Leica Biosystems, Deer Park, IL, USA), followed by paraffin embedding using HistoCore Arcadia Embedding Center (Leica Biosystems, Deer Park, IL, USA). Paraffin blocks were cut into 55-μm-thick sections with HistoCore BIOCUT—Manual Rotary Microtome (Leica Biosystems, Deer Park, IL, USA). Aortic samples were mounted on a glass slide and stained as described below for elastin and collagen analysis.

### 2.9. Collagen Detection

Aortic collagen levels were evaluated using the Picrosirius staining kit (Abcam, Cambridge, MA, USA, ab150681), following the manufacturer’s protocol. Images were obtained in a Zeiss Model AxioSkop-2 MOT microscope (10× magnification) and later analyzed using ImageJ software version 1.54p (NIH, Bethesda, MD, USA) to measure the total collagen (using % of positive area with red stain) in the whole ring (including media + adventitia layer) and the middle layer.

### 2.10. Elastin

Aortic sections embedded in paraffin-embedded sections were deparaffinized in Histoclear reagent and dehydrated in graded ethanol in series (100%, 95%, and 70%). Samples were stained for elastin using the Verhoeff Elastic technique with an Elastic stain kit (Epredia, Kalamazoo, MI, USA, No. 87017), following the instructions provided by the manufacturer’s instructions. The images were obtained in a Zeiss Model AxioSkop-2 MOT microscope (10× magnification). The elastin content was quantified using ImageJ Software at 10× magnification using the area function. Additionally, to evaluate elastin fragmentation, the region of interest was chosen based on optical images to include the areas showing elastin fibers and was captured using AFM.

### 2.11. Data Analysis

Graphs were prepared using the GraphPad Prism software 10.3.1 (GraphPad Software, San Diego, CA, USA). Values are reported as means ± SEM. One-way analysis of variance (ANOVA) with Bonferroni’s post hoc test was used to evaluate the data, and Grubb’s test was used to check for any outliers. All data points for each experiment group were included. The results were considered statistically significant at a probability value of *p* ≤ 0.05, and (n) represents the number of animals per group.

## 3. Results

### 3.1. Losartan Treatment Improves the Animal Profile in Hypertensive Females

**Figure 1:**
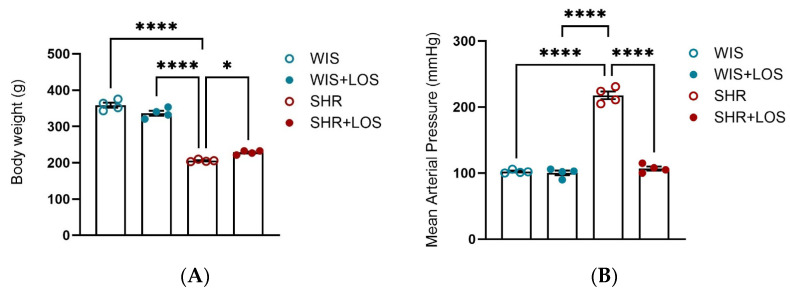
Effect of losartan on (**A**) Body-weight and (**B**) Mean Arterial Pressure. WIS (blue) and SHR (red), treated (solid symbols) and untreated (open symbols). Values are reported as mean ± SEM. One-way ANOVA followed by Bonferroni’s post hoc was used for comparison. Probability considered significant: * *p* < 0.05, **** *p* < 0.0001; n = 4 per group.

The hypertensive profile was confirmed, and losartan treatment improved both the body weight and mean arterial blood pressure in the middle-aged female SHRs compared with their untreated normotensive group (Figure 1A,B).

### 3.2. Hypercontractility to PE in the Aorta of Middle-Aged Hypertensive Female Rats Was Restored by Losartan Treatment

**Figure 2:**
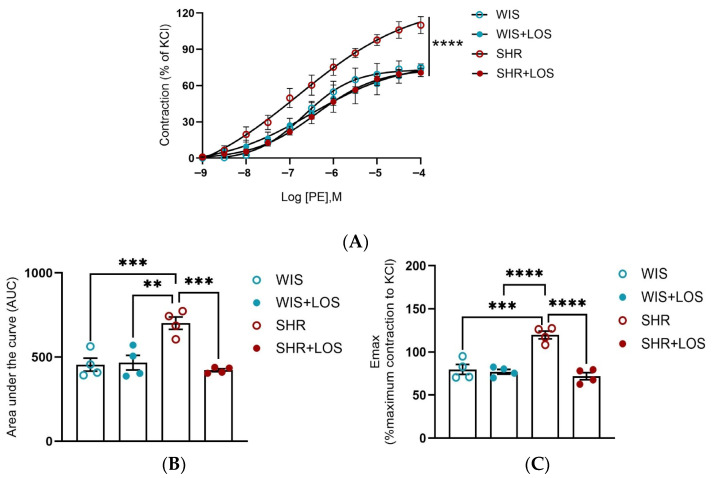
Effect of losartan treatment on the vascular contraction in response to adrenergic stimulation. (**A**) Cumulative concentration–response curve to Phenylephrine (PE). (**B**) Area under the curve to PE curve (AUC). (**C**) Emax of the PE contraction normalized by KCl. WIS (blue) and SHR (red), treated (solid symbols) and untreated (open symbols). Values are reported as mean ± SEM. One-way ANOVA followed by Bonferroni’s post hoc was used for comparisons. Probability considered significant: ** *p* < 0.001, *** *p* < 0.001, and **** *p* < 0.0001; n = 4 per group.

Hypertension-related vascular dysfunction is associated with increased vasoconstriction. To confirm this, a cumulative-concentration response curve to PE was performed, and SHR females displayed increased force development compared with WIS controls (Figure 2A–C). Treatment with losartan significantly decreased and restored this hyperresponsiveness to adrenergic stimulation in the aorta of middle-aged SHR females (Figure 2A). Quantification of contractile responses by area under the curve (AUC) and maximum contraction (Emax) confirmed these findings, showing that losartan attenuated the heightened vasoconstriction observed in SHRs (Figure 2B,C).

### 3.3. Losartan Treatment Attenuates Ca^2+^ Mishandling in the Aorta of Middle-Aged Hypertensive Females

**Figure 3:**
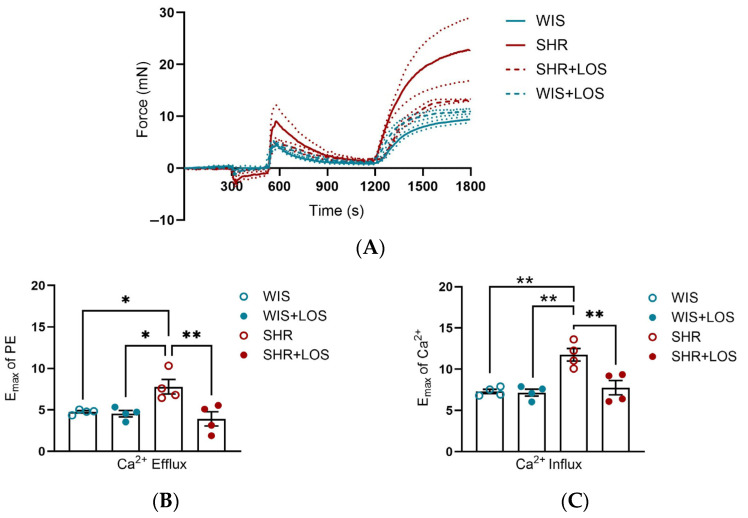
Calcium dynamic-mediated vascular contraction in the aorta of hypertensive rats. (**A**) Representative time vs. force curves in the calcium protocol of the aortas of hypertensive female rats. (**B**) Emax in response to PE, reflecting calcium efflux. (**C**) Emax following Ca^2+^, reflecting calcium influx. WIS (blue) and SHR (red), treated (solid symbols) and untreated (open symbols). Values are reported as mean ± SEM. One-way ANOVA followed by Bonferroni’s post hoc was used for comparisons. Probability considered significant: * *p* < 0.05 and ** *p* < 0.01; n = 4 per group.

To evaluate the effects of losartan on Ca^2+^ dynamics, we performed functional experiments in which aortic rings were placed initially in Ca^2+^-free PSS and subsequently challenged with a single dose of PE. After evaluating the force of contraction for 10 min, extracellular Ca^2+^ was restored (at the time 1200 s), and the resulting force was evaluated for 10 min. Middle-aged SHR females exhibited greater efflux (Figure 3A,B) and influx of Ca^2+^ (Figure 3A,C) compared to WIS females, which was attenuated by losartan treatment (Figure 3A–C). This data suggests that losartan improves the Ca^2+^ mishandling involving the sarcoplasmic reticulum and plasmalemmal membrane sources of Ca^2+^, resulting in a lower degree of contraction in the aorta of middle-aged SHR females.

### 3.4. Losartan Ameliorates the Phasic and Tonic Components of the Contraction Curve in the Hypertensive Aorta

**Figure 4:**
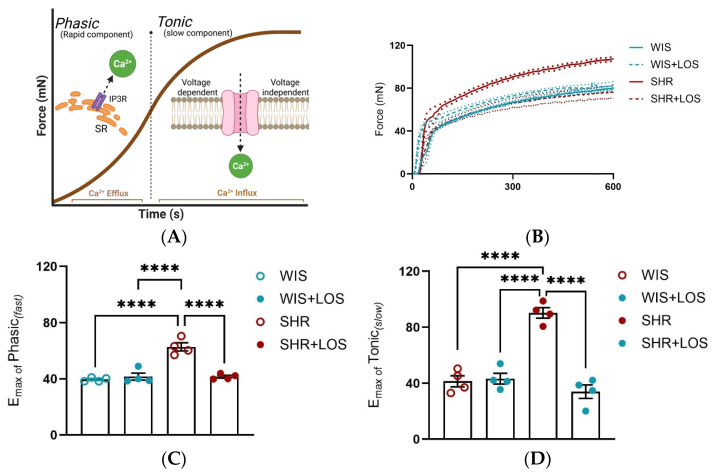
Effect of losartan on the components of the biphasic contraction curve. (**A**) Schematic of the mechanism underlying Ca^2+^ efflux during phasic and Ca^2+^ influx during tonic contraction components. (**B**) Representative time vs. force curve in response to a single dose of PE in the aorta of hypertensive females. (**C**) Emax of Phasic Contraction. (**D**) Emax of Tonic Contraction. WIS (blue) and SHR (red), treated (dashed line and solid symbols) and untreated (continuous line and open symbols). Values are reported as mean ± SEM. One-way ANOVA followed by Bonferroni’s post hoc was used for comparisons. Probability considered significant: **** *p* < 0.0001; n = 4 per group.

To confirm Ca^2+^ mishandling, stimulation with an α-1 adrenergic agonist (PE), which leads to the formation of a biphasic contraction curve in the aorta, was performed. This curve consists of a phasic component (fast) due to Ca^2+^ release from sarcoplasmic reticulum and a tonic component (slow) driven by Ca^2+^ influx from plasmalemmal channels. We exposed aortic rings to a single dose of PE (10 μmol/L) and measured the force development generated each second for 10 min. In aortas from female SHRs, both phasic and tonic contraction components were increased, resulting in greater force generation, which was abolished by losartan treatment compared to the non-treated SHR group (Figure 4A–C), reinforcing that losartan treatment restores Ca^2+^ handling mechanisms in this tissue.

### 3.5. Losartan Reduced Free Intracellular Ca^2+^ Levels in the Hypertensive Aorta of Middle-Aged Females

**Figure 5:**
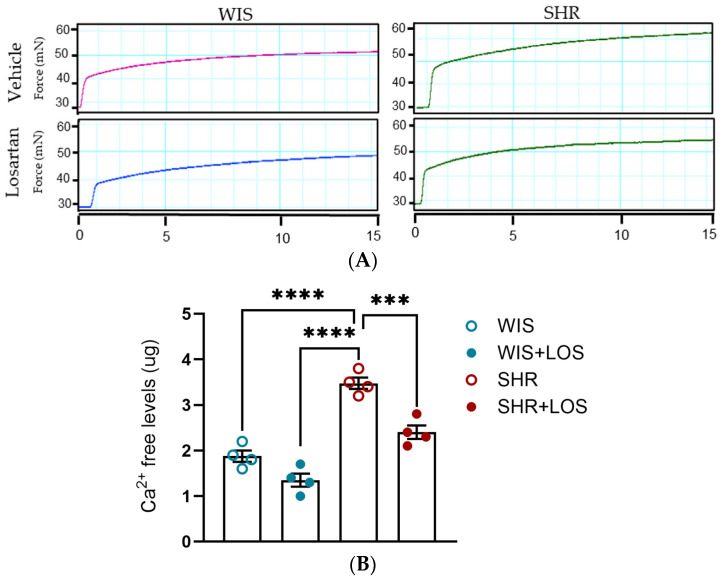
Effect of losartan on free intracellular Ca^2+^ levels. (**A**) Representative time–force curves in the aortas of hypertensive females. (**B**) Levels of intracellular Ca^2+^. WIS (blue) and SHR (red), treated (solid symbols) and untreated (open symbols). Values are reported as mean ± SEM. One-way ANOVA followed by Bonferroni’s post hoc was used for comparisons. Probability considered significant: *** *p* < 0.001 and **** *p* < 0.0001; n = 4 per group.

Considering that the vasculature relies on cytosolic Ca^2+^ levels for contraction, the levels of free Ca^2+^ were assessed in the aortic rings stimulated with a single dose of PE (10 μmol/L) for 15 min (Figure 5A). The SHR aorta exhibited increased levels of intracellular Ca^2+^ compared to the WIS group, which was significantly reduced with losartan treatment (Figure 5B).

### 3.6. Losartan Restored Pulse Wave Velocity and Hemodynamic Parameters in the Aorta of Middle-Aged SHR Females

**Figure 6:**
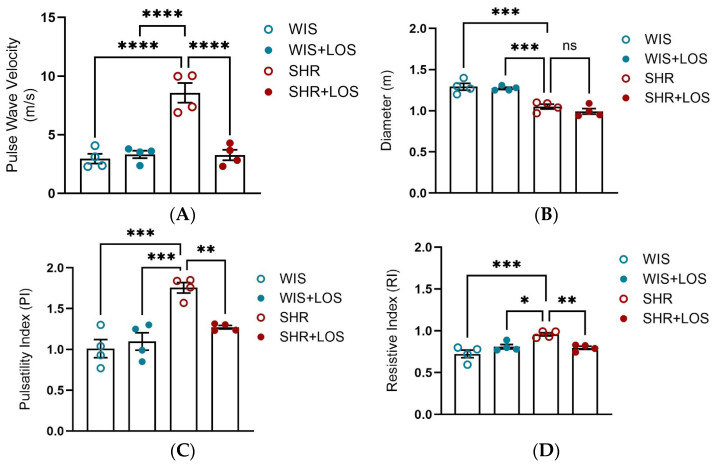
In vivo assessment of aortic stiffness and hemodynamic parameters using echocardiography following losartan treatment. (**A**) Aortic PWV. (**B**) Aortic diameter (m). (**C**) Pulsatility Index (PI) and (**D**) Resistive Index (RI). WIS (Blue) and SHR (red), treated (solid symbols) and untreated (open symbols). Values are reported as mean ± SEM. One-way ANOVA followed by Bonferroni’s post hoc was used for comparisons. Probability considered significant: * *p* < 0.05, ** *p* < 0.01, *** *p* < 0.001, and **** *p* < 0.0001; n = 4 per group.

To evaluate the effect of losartan treatment on stiffness at the macro-level, in vivo PWV and hemodynamic parameters were evaluated using echocardiography. SHR females exhibited significantly higher PWV compared to normotensive WIS females, which was markedly reduced following losartan treatment, restoring values close to WIS levels (Figure 6A). Aortic diameter was decreased in SHR compared to WIS, and it was not affected by losartan treatment (Figure 6B). In addition, SHR females displayed higher PI and RI compared to WIS, reflecting impaired compliance. Treatment with losartan attenuated both indices (Figure 6C,D).

### 3.7. Losartan Prevented Augmented Stiffness in the Aorta of Middle-Aged SHR Females

**Figure 7:**
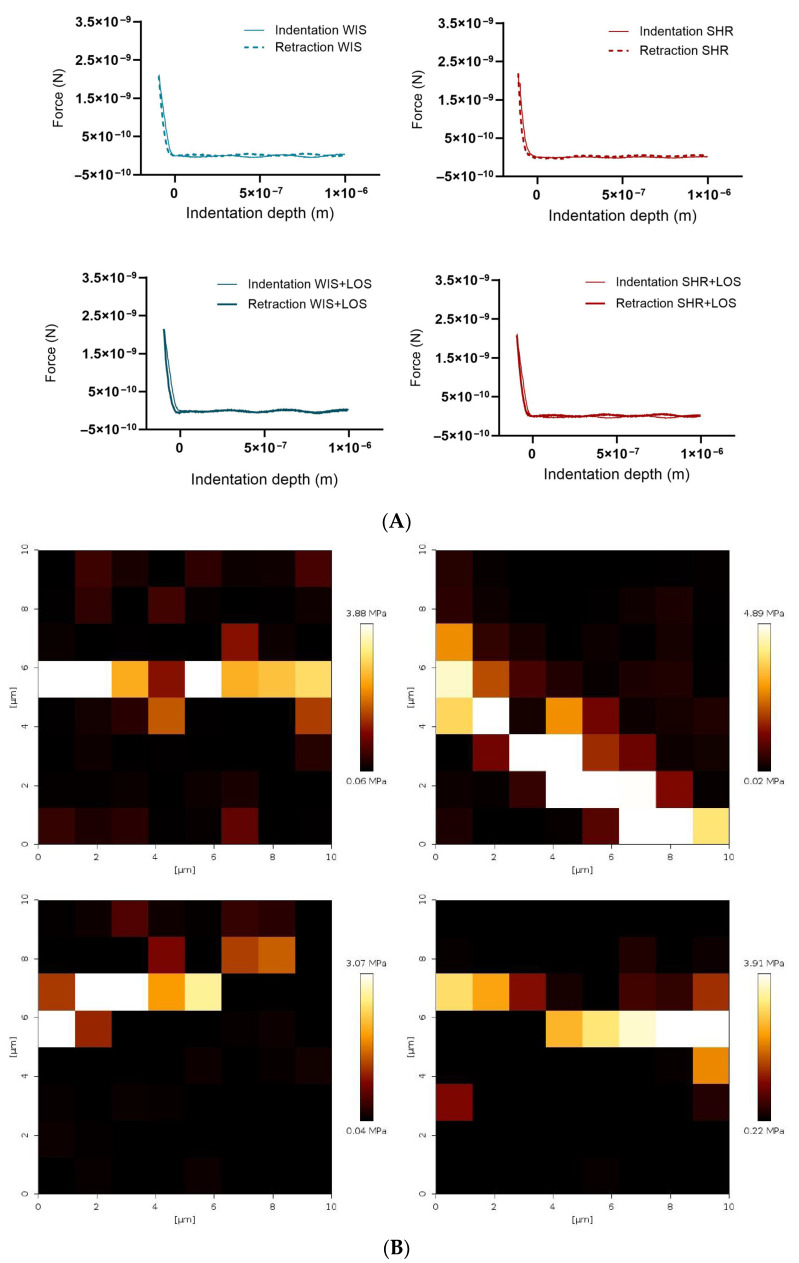

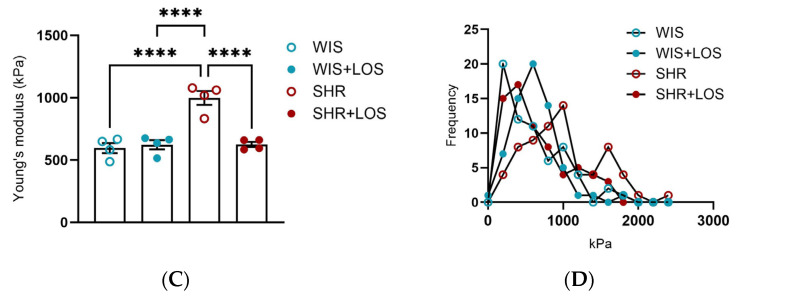
Effect of losartan on the aortic stiffness of middle-aged hypertensive females detected at the nano-level. (**A**) Representative force–indentation curves in the aorta of hypertensive females. (**B**) Stiffness map for the samples. (**C**) Averaged Young’s modulus. (**D**) Histogram showing frequency of measured stiffness. WIS (Blue) and SHR (red), treated (solid symbols) and untreated (open symbols) middle-aged females. Values are reported as mean ± SEM. One-way ANOVA followed by Bonferroni’s post hoc was used for comparisons. Probability considered significant: **** *p* < 0.0001; n = 4 per group.

To further confirm stiffness and biomechanical changes, aortic rings were evaluated at the nano-level using AFM. Nano-indentation was performed on a 10 × 10 µm^2^ area using a probe with a 20 nm tip radius, and the resulting force–indentation curves were processed to calculate stiffness values at each indentation point (Figure 7A). AFM stiffness mapping further demonstrated regional differences across groups (Figure 7B). We reported that hypertension increased aortic medial layer stiffness in middle-aged SHR females, as shown by Young’s modulus, which is restored with the losartan treatment (Figure 7C). The evident rightward shift in the untreated SHR group in the distribution of the Young’s modulus suggests increased heterogeneity in vessel wall stiffness observed in middle-aged SHR females, which was improved following losartan treatment in the treated group (Figure 7D).

### 3.8. Losartan Treatment Restores Collagen and Elastin Content in the Aorta of Middle-Aged SHR Females

**Figure 8:**
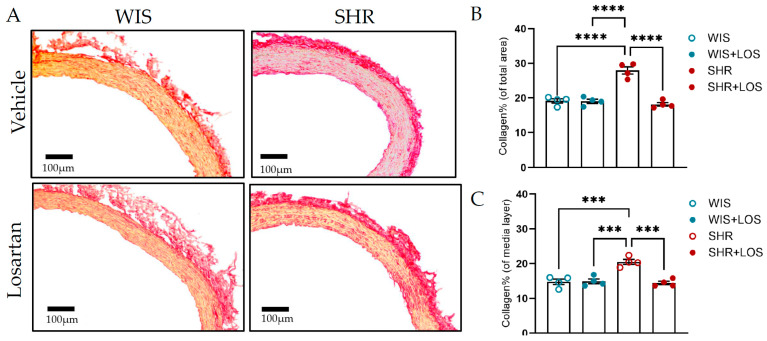

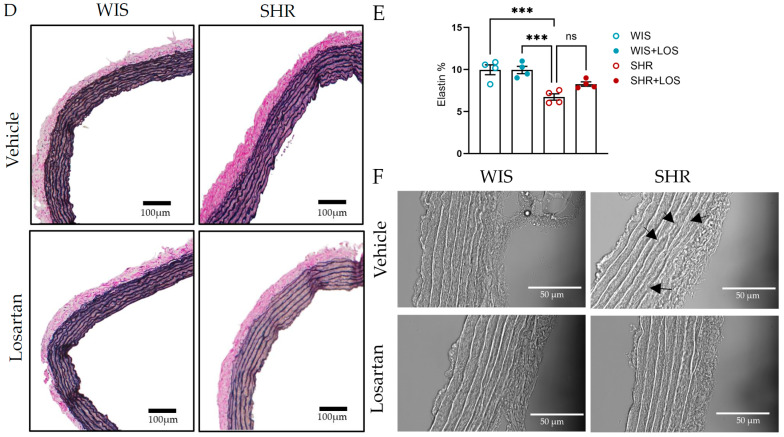
Impact of losartan on aortic ECM components, collagen, and elastin. (**A**) Picrosirius stain of collagen (scale bar: 100 μm; collagen stained in red). (**B**) Collagen % of the total vessel area. (**C**) Collagen % of the media layer. (**D**) Elastin by Van Gieson stain (scale bar: 100 μm). (**E**) Elastin content. (**F**) Image obtained from AFM, indicating elastin fragmentation in the SHR group (arrow; scale bar: 50 μm) in the aorta. WIS (blue) and SHR (red), treated (solid symbols) and untreated (open symbols). Values are reported as mean ± SEM. One-way ANOVA followed by Bonferroni’s post hoc was used for comparisons. Probability considered significant: *** *p* < 0.001 and **** *p* < 0.0001; n = 4 per group.

ECM is the non-cellular component of the vessel wall that provides biochemical support, primarily through collagen and elastin [13]. Hypertension promotes vascular remodeling by disrupting the organization of these proteins. To measure these changes, collagen and elastin were assessed indirectly using Picrosirius and Van Gieson’s staining kit, respectively. Compared with the aorta from normotensive WIS females, the SHR aorta exhibited increased collagen deposition, with collagen fibers stained red (Figure 8A), throughout the vessel and in the medial layer, which was significantly reduced following treatment with losartan, as shown by analyzing collagen content in the total area and in the medial layer, which reflects the VSMC (Figure 8B,C). In contrast, SHR aortic sections displayed decreased elastin content compared to WIS, as shown in images stained blue (Figure 8D). Losartan treatment showed a trend toward restoring elastin, though the difference was not statistically significant (Figure 8E). Notably, elastin fragmentation present in the aorta of middle-aged female SHR was no longer noted following the treatment with losartan when observed in the AFM (Figure 8F; pointed with arrows). Together, these findings suggest that losartan preserves aortic structural integrity, contributing to mitigating vascular dysfunction in hypertension.

## 4. Discussion

Hypertension remains one of the leading causes of cardiovascular morbidity and mortality worldwide, yet sex- and age-related differences in vascular function are often underexplored. While females generally exhibit lower blood pressure (BP) than males at a younger age [27], middle-aged females show a much steeper rise in BP and a greater incidence of hypertension-related vascular dysfunction, as estrogen-mediated protection declines [4,5]. Moreover, antihypertensive treatment responses differ by sex, with females often showing higher drug exposure and greater susceptibility to adverse effects [28]. Clinical outcomes also differ, as females treated with angiotensin receptor blockers (ARBs) have demonstrated better survival compared to males [29]. Among ARBs, losartan is widely prescribed, and emerging data suggest females may be particularly responsive to losartan, with lower cardiovascular event rates compared to males [13,14].

Despite evidence of sex-based differences in hypertension and its treatment, it remains unclear whether losartan can ameliorate vascular dysfunction in the aorta of middle-aged hypertensive females by stabilizing Ca^2+^ mishandling, which is a key component driving vascular dysfunction in hypertension. To address this gap, we investigated the effects of losartan treatment on the aorta of female SHRs. Our findings demonstrated that long term treatment with losartan: (1) lowers mean arterial pressure and reduces aorta hypercontractility, (2) normalizes abnormalities in Ca^2+^ dynamics in the aorta by stabilizing Ca^2+^ efflux and influx into VSMC, (3) prevents abrupt increase in free intracellular Ca^2+^ in the vasculature, (4) improves aortic stiffness and hemodynamic parameters, and (5) mitigates ECM remodeling by restoring collagen and elastin content. Together, our results suggest losartan plays an important role in reestablishing Ca^2+^ dynamics in the vasculature of the middle-aged hypertensive female, and support that AT1R blockers’ benefits might extend beyond only lowering BP, especially in females.

First, we confirmed characteristics of the hypertensive model and the impact of losartan on MAP. Compared to age-matched WIS, SHR females exhibited elevated MAP and reduced body weight (Figure 1A,B), consistent with the hypertensive phenotype described for the SHR model, which develops elevated MAP with age and mimics human essential hypertension [30,31,32]. Chronic treatment with losartan significantly reduced MAP and improved body weight as expected (Figure 1A,B).

Since hypercontractility is a hallmark of hypertension with functional consequences in the vasculature, and the SHR model is also characterized by increased sensitivity of aortic smooth muscle alpha receptors [33], we evaluated PE-induced dose responses in aortic rings of hypertensive females and the impact of losartan treatment. Our results show a heightened vasoconstriction response to PE in middle-aged female SHRs compared to WIS (Figure 2A), confirming that PE-induced hypercontractility persists into middle age during hypertertension, a condition where vascular dysfunction is further exacerbated by aging. Chronic treatment with losartan significantly reduced this hypercontractility, restoring both Emax and AUC to control levels (Figure 2A–C).

The vascular tone is tightly regulated by intracellular Ca^2+^ levels in VSMCs, and hypertension is associated with altered Ca^2+^ handling [9]. Currently, many new pathways associated with Ca^2+^ mishandling in the vasculature have emerged [34,35]. Therefore, we examined whether chronic treatment with losartan improves Ca^2+^ mishandling in the aorta of middle-aged hypertensive females. We found that aortas from untreated SHR exhibited significantly increased Ca^2+^ efflux from the SR and Ca^2+^ influx from extracellular sources (Figure 3), corroborating disrupted Ca^2+^ dynamics as a central component of hypertensive vasculature. This finding aligns with earlier studies showing that VSMCs from SHR exhibit elevated basal and Ang II-stimulated intracellular Ca^2+^ levels in both sexes [36]. Additionally, studies of TRPC (Transient Receptor Potential Channel)-Ca^2+^ entry (SOCE) and Na^+^/Ca^2+^ exchanger activity in male SHR demonstrated altered influx and efflux in the vasculature [37,38,39]. Notably, sex differences in Ca^2+^ regulation are well-documented, with female SHR exhibiting reduced contraction to PE and KCl-induced Ca^2+^ entry compared to males, partly linked to estrogen signaling [11,12]. However, most of the research on this topic was focused on younger or male SHR, leaving a gap in our understanding of Ca^2+^ mishandling in middle-aged females. Importantly, the effects of losartan, the first-line medication for treating hypertension, include cardiovascular protection. Yet, sex- and age-specific studies are needed for a better-tailored treatment.

Prior works with losartan showed this drug significantly reduces intracellular free Ca^2+^ in aortic VSMCs [40], inhibits phosphoinositide signaling in VSMCs of normotensive WKY rats [41], and is more effective than a Ca^2+^ channel blocker in reducing BP, endothelin-1 (ET-1), and vasoconstriction in the aorta [42]. Building on this, we investigated whether losartan treatment could prevent the Ca^2+^ mishandling in the aorta of hypertensive female middle-aged. Indeed, our results showed that chronic treatment with losartan normalized both the Ca^2+^ efflux and influx (Figure 3B,C) in this condition, highlighting its ability to restore Ca^2+^ homeostasis.

To further confirm the disrupted Ca^2+^ behavior, we examined the biphasic nature of vascular contraction. Both the phasic component (rapid, sarcoplasmic reticulum-mediated Ca^2+^ release) and the tonic component (sustained, extracellular Ca^2+^-dependent influx) were significantly elevated in SHR compared with WIS (Figure 4B,C). While physiological studies have reported reduced phasic and tonic force in middle-aged male rats [43], our study is the first to show that both components are enhanced in the aorta of middle-aged hypertensive females. Importantly, chronic treatment with losartan not only significantly reduced overall force generation (Figure 4A) but also restored both phasic and tonic responses to the levels of normotensive WIS (Figure 4B,C). Corroborating our results, prior work has shown that losartan decreases PE-induced contraction in the aorta of SHR, likely through the endothelium-dependent mechanisms involving nitric oxide [44]. Rodrigues et al. demonstrated that losartan improves endothelial dysfunction largely through increased vasodilatory responses and Angiotensin II Type 2 (AT2) receptor expression in females. Although their work focused on relaxation rather than contraction, it reinforces the concept that losartan exerts sex-specific protective effects on vascular reactivity [45]. Our results extend these findings by revealing that losartan reverses hypercontractility via restoring proper Ca^2+^ handling and re-establishing normal vascular tone in middle-aged females.

To further validate our findings on the effects of losartan on Ca^2+^ handling, we measured free intracellular Ca^2+^ levels in aortic tissue using a biochemical approach. We observed higher basal levels of Ca^2+^ in middle-aged female SHRs compared to WIS controls (Figure 5), consistent with elevated intracellular Ca^2+^ in the vasculature leading to hypercontractility. Corroborating our results, Bhalla et al. demonstrated an age-dependent increase in vascular Ca^2+^ content in SHR compared to their matched normotensive counterparts, even though differences were not observed at early ages (4–5 weeks) [46]. Conversely, Nabika et al. showed that Ca^2+^ levels from cultured VSMCs of SHR and Wistar Kyoto (WKY) rats were not different from each other [47], highlighting the complexity of interpreting total tissue Ca^2+^ measurements in terms of transmembrane fluxes or compartmental distribution. We reported that treatment with losartan markedly reduced intracellular Ca^2+^ concentrations in the aortas of SHR to levels almost at those of normotensive WIS (Figure 5B).

However, vascular dysfunction in hypertension is not limited to impaired contractility associated with Ca^2+^ behavior; it also involves changes in the structural properties of arteries. Stiffness in large arteries is a hallmark of essential hypertension, as chronic elevations in BP induce vascular wall remodeling, primarily through hypertrophy, compromising vascular compliance [48,49,50,51]. As the main conduit artery, the aorta regulates BP through two distinct functions: (1) as a conduit, ensuring delivery of blood to peripheral organs, and (2) as a cushion, which dampens pulsatile pressure and shapes the pulse wave [52]. This ability, known as the Windkessel function [52], allows the aorta to maintain pulsatility, which makes it important to study. However, in hypertension, the early return of the reflected pulse wave during systole elevates systolic pressure, disrupts ventricular–vascular coupling, and exacerbates aortic stiffness [52]. Given our focus on vasculature at macro-, micro-, and nano-levels, characterizing both structural and functional alterations of the aorta is essential for understanding hypertension-associated vascular dysfunction, as well as elucidating whether losartan reduces stiffness in an age- and sex-dependent manner.

To assess stiffness in vivo and cardiovascular parameters, we used PWV and echocardiography. In our study, middle-aged SHR females displayed increased PWV (Figure 6A) and impaired compliance indices, accompanied by elevated Resistive (RI) and Pulsatility (PI) indices (Figure 6C,D), while aortic diameter was reduced compared to controls (Figure 6B). Similar trends have been reported in male SHR, where PWV rises from young to old rats, reflecting an increase in stiffness with disease progression and age [53,54]. Chronic treatment with losartan attenuated the elevated stiffness (Figure 6A) and improved RI and PI but had no effect on aortic diameter (Figure 6B). This observation is consistent with reports that ARBs effectively reduce medial thickness and the media-lumen ratio without significantly altering lumen diameter [55,56]. This data suggests that losartan can improve resistance and vascular compliance, even when no change in aortic diameter occurs. In contrast, a clinical study displayed no significant effect of losartan on RI and PI [57], indicating possible differences related to species, sex, or treatment duration.

AFM was employed here to characterize the mechanical properties and topography of the aortic structure at the nano-level, and confirm the stiffness observed in vivo. Results showed increased medial layer stiffness as indicated by higher Young’s modulus, which was prevented by losartan (Figure 7C). To the best of our knowledge, this is the first demonstration of losartan-mediated improvement in medial stiffness of middle-aged SHR females. Collectively, our data highlights that biomechanical alterations contributing to macroscopic vascular changes can be prevented following losartan treatment.

Building on our findings, we next examined the structural basis of these biomechanical alterations, focusing on the main components of arterial ECM, collagen, and elastin. In physiological conditions, sex-associated functional differences in aortas are linked to differences in the dynamics of vascular reactivity and ECM composition [58]. During hypertension, vascular dysfunction drives ECM remodeling- caused by a shift in collagen and elastin balance, which increases aortic stiffness and cardiovascular risk [48,49,50,51]. Clearly, our histological analysis revealed marked collagen accumulation in the medial and total vessel wall, in addition to elastin degradation and fragmentation (Figure 8), consistent with the ECM remodeling characteristics of hypertension [49]. Losartan partially reversed these organizational changes in the ECM, reducing collagen (Figure 8B,C) and limiting elastin fragmentation (Figure 8F), though total elastin content was not significantly restored (Figure 8E). The observed trend towards improvement suggests a protective effect against progressive ECM deterioration in line with studies showing the effectiveness of ARBs in reversal of ECM remodeling in conductive and resistance arteries during hypertension in males [51,55]. Interestingly, reports on elastin remain variable: some studies show increased elastin in SHR and other hypertension models [59,60,61] as a compensatory mechanism, while others report reduced elastin or fragmentation [55,62]. This discrepancy reflects differences in vascular beds, disease progression, and age. Mechanistically, collagen remains vulnerable to fibrosis while elastin’s production is limited, as its synthesis largely ceases by adulthood, making it more prone to increased proteolytic degradation under hypertension [63,64]. Losartan thus likely preserves elastin integrity and may be reducing breakdown activity rather than inducing active elastin synthesis.

It is important to acknowledge a few limitations encountered in this study. While the focus of the study was on females, including male rats could have provided insights into responsiveness to losartan with age progression between sexes. Additionally, although we demonstrated functional, biomechanical, and structural changes in the aorta, we did not directly measure Ca^2+^ activity using techniques such as patch clamp. Nevertheless, the combination of techniques that unveil functional, biomechanical, and structural changes from macro- to nano-levels provides a sophisticated and comprehensive overview of the effects of losartan in the aortas of middle-aged hypertensive females.

## 5. Conclusions

In conclusion, our study demonstrates that losartan normalizes Ca^2+^ mobilization, attenuates hypercontractility, reduces stiffness, and mitigates ECM remodeling in the aorta of middle-aged female SHRs. These results highlight Ca^2+^ mishandling as a central driver of female vascular dysfunction, underscoring losartan as a promising treatment for preserving vascular Ca^2+^ dynamics, function, and structure in females.

## Data Availability

The original contributions presented in the study are included in the article/Supplementary Material; further inquiries can be directed to the corresponding authors.

## Supplementary Materials

The following supporting information can be downloaded at: https://www.mdpi.com/article/10.3390/jcdd12110441/s1, Table S1: Statistical analysis and *p*-values for the body weight and blood pressure of the middle-aged hypertensive females; Table S2: Statistical analysis and *p*-values for Area Under the Curve (AUC) and Emax for the Concentration-Response curve in the aorta of middle-aged hypertensive females; Table S3: Statistical analysis and *p*-values for Ca^2+^ protocol in th aorta of middle-aged hypertensive females; Table S4: Statistical analysis and *p*-values for phasic and tonic components of biphasic contraction in the aorta of middle-aged hypertensive females; Table S5: Statistical analysis and *p*-values for intracellular Ca^2+^ levels in the aorta of middle-aged hypertensive females; Table S6: Statistical analysis and *p*-values for the PWV, Diameter, Pulsatility Index, Resistivity Index in the aorta of middle-aged hypertensive females; Table S7: Statistical analysis and *p*-values for the Young’s modulus in the aorta of middle-aged hypertensive females; Table S8: Statitical analysis and *p*-values for the Collagen % of the total vessel area, Collagen % of the media layer and Elastin % in the aorta of middle-aged hypertensive females.

## Abbreviations

The following abbreviations are used in this manuscript:

VSMCs	Vascular Smooth Muscle Cells
CVD	Cardiovascular disease
MAP	Mean Arterial Pressure
AngII	Angiotensin II
ARB	Angiotensin II Receptor Blocker
SHR	Spontaneously Hypertensive Rats
WIS	Wistar
AT1R	Angiotensin I Receptor Blocker
PE	Phenylephrine
PWV	Pulse Wave Velocity
AFM	Atomic Force Microscopy
PI	Pulsatility Index
RI	Resistive Index

## References

[1] Mills K.T., Bundy J.D., Kelly T.N., Reed J.E., Kearney P.M., Reynolds K., Chen J., He J. (2016). Global Disparities of Hypertension Prevalence and Control: A Systematic Analysis of Population-Based Studies From 90 Countries. Circulation.

[2] Iqbal A.M., Jamal S.F. (2025). Essential Hypertension. StatPearls.

[3] Kario K., Okura A., Hoshide S., Mogi M. (2024). The WHO Global report 2023 on hypertension warning the emerging hypertension burden in globe and its treatment strategy. Hypertens. Res. Off. J. Jpn. Soc. Hypertens..

[4] Ji H., Kim A., Ebinger J.E., Niiranen T.J., Claggett B.L., Bairey Merz C.N. (2020). Sex Differences in Blood Pressure Trajectories Over the Life Course. JAMA Cardiol..

[5] Hildreth K.L., Ozemek C., Kohrt W.M., Blatchford P.J., Moreau K.L. (2018). Vascular dysfunction across the stages of the menopausal transition is associated with menopausal symptoms and quality of life. Menopause.

[6] Kwan C.Y. (1985). Dysfunction of calcium handling by smooth muscle in hypertension. Can. J. Physiol. Pharmacol..

[7] Kuo I.Y., Ehrlich B.E. (2015). Signaling in muscle contraction. Cold Spring Harb. Perspect. Biol..

[8] Fransen P., Van Hove C.E., Leloup A.J., Martinet W., De Meyer G.R., Lemmens K., Bult H., Schrijvers D.M. (2015). Dissecting out the complex Ca^2+^-mediated phenylephrine-induced contractions of mouse aortic segments. PLoS ONE.

[9] Dai C., Khalil R.A. (2025). Calcium Signaling Dynamics in Vascular Cells and Their Dysregulation in Vascular Disease. Biomolecules.

[10] Harraz O.F., Jensen L.J. (2021). Vascular calcium signalling and ageing. J. Physiol..

[11] Crews J.K., Murphy J.G., Khalil R.A. (1999). Gender differences in Ca^2+^ entry mechanisms of vasoconstriction in Wistar-Kyoto and spontaneously hypertensive rats. Hypertension.

[12] Murphy J.G., Khalil R.A. (2000). Gender-specific reduction in contractility and [Ca^2+^] in vascular smooth muscle cells of female rat. Am. J. Physiol. Physiol..

[13] Silva-Antonialli M., Fortes Z., Carvalho M., Scivoletto R., Nigro D. (2000). Sexual dimorphism in the response of thoracic aorta from SHRs to losartan. Gen. Pharmacol. Vasc. Syst..

[14] Os I., Franco V., Kjeldsen S.E., Manhem K., Devereux R.B., Gerdts E., Hille D.A., Lyle P.A., Okin P.M., Dahlöf B. (2008). Effects of losartan in women with hypertension and left ventricular hypertrophy: Results from the Losartan Intervention for Endpoint Reduction in Hypertension Study. Hypertension.

[15] Zhu Z., Zhang S.H., Wagner C., Kurtz A., Maeda N., Coffman T., Arendshorst W.J. (1998). Angiotensin AT1B receptor mediates calcium signaling in vascular smooth muscle cells of AT1A receptor-deficient mice. Hypertension.

[16] Samain E., Bouillier H., Perret C., Safar M., Dagher G. (1999). ANG II-induced Ca^2+^ increase in smooth muscle cells from SHR is regulated by actin and microtubule networks. Am. J. Physiol. Circ. Physiol..

[17] Kilkenny C., Browne W.J., Cuthill I.C., Emerson M., Altman D.G. (2010). Improving bioscience research reporting: The ARRIVE guidelines for reporting animal research. PLoS Biol..

[18] Jones D.W., Ferdinand K.C., Taler S.J., Johnson H.M., Shimbo D., Abdalla M., Altieri M.M., Bansal N., Bello N.A., Writing Committee Members (2025). 2025 AHA/ACC/AANP/AAPA/ABC/ACCP/ACPM/AGS/AMA/ASPC/NMA/PCNA/SGIM Guideline for the Prevention, Detection, Evaluation and Management of High Blood Pressure in Adults: A Report of the American College of Cardiology/American Heart Association Joint Committee on Clinical Practice Guidelines. Circulation.

[19] McEvoy J.W., McCarthy C.P., Bruno R.M., Brouwers S., Canavan M.D., Ceconi C., Christodorescu R.M., Daskalopoulou S.S., Ferro C.J., Gerdts E. (2024). 2024 ESC Guidelines for the management of elevated blood pressure and hypertension: Developed by the task force on the management of elevated blood pressure and hypertension of the European Society of Cardiology (ESC) and *endorsed by the European Society of Endocrinology (ESE) and the European Stroke Organisation (ESO)*. Eur. Heart J..

[20] Soltis E.E. (1993). Alterations in vascular structure and function after short-term losartan treatment in spontaneously hypertensive rats. J. Pharmacol. Exp. Ther..

[21] Maeso R., Rodrigo E., Muñoz-García R., Navarro-Cid J., Ruilope L.M., Cachofeiro V., Lahera V. (1998). Chronic treatment with losartan ameliorates vascular dysfunction induced by aging in spontaneously hypertensive rats. J. Hypertens..

[22] Cerbai E., Crucitti A., Sartiani L., De Paoli P., Pino R., Rodriguez M.L., Gensini G., Mugelli A. (2000). Long-term treatment of spontaneously hypertensive rats with losartan and electrophysiological remodeling of cardiac myocytes. Cardiovasc. Res..

[23] de Oliveira A.A., Priviero F., Tostes R.C., Webb R.C., Nunes K.P. (2021). Dissecting the interaction between HSP70 and vascular contraction: Role of Ca^2+^ handling mechanisms. Sci. Rep..

[24] Lee L., Cui J.Z., Cua M., Esfandiarei M., Sheng X., Chui W.A., Xu M.H., Sarunic M.V., Beg M.F., van Breemen C. (2016). Aortic and Cardiac Structure and Function Using High-Resolution Echocardiography and Optical Coherence Tomography in a Mouse Model of Marfan Syndrome. PLoS ONE.

[25] Wu W., Xie M., Qiu H. (2021). The Progress of Advanced Ultrasonography in Assessing Aortic Stiffness and the Application Discrepancy between Humans and Rodents. Diagnostics.

[26] Bradley T.J., Potts J.E., Potts M.T., DeSouza A.M., Sandor G.G. (2005). Echocardiographic Doppler assessment of the biophysical properties of the aorta in pediatric patients with the Marfan syndrome. Am. J. Cardiol..

[27] Safar M.E., Smulyan H. (2004). Hypertension in women. Am. J. Hypertens..

[28] Soldin O.P., Mattison D.R. (2009). Sex differences in pharmacokinetics and pharmacodynamics. Clin. Pharmacokinet..

[29] Hudson M., Rahme E., Behlouli H., Sheppard R., Pilote L. (2007). Sex differences in the effectiveness of angiotensin receptor blockers and angiotensin converting enzyme inhibitors in patients with congestive heart failure—A population study. Eur. J. Heart Fail..

[30] Yamori Y. (1977). Pathogenesis of spontaneous hypertension as a model for essential hypertension. Jpn. Circ. J..

[31] Lerman L.O., Kurtz T.W., Touyz R.M., Ellison D.H., Chade A.R., Crowley S.D., Mattson D.L., Mullins J.J., Osborn J., Eirin A. (2019). Animal Models of Hypertension: A Scientific Statement From the American Heart Association. Hypertension.

[32] Safar M., Chamiot-Clerc P., Dagher G., Renaud J.F. (2001). Pulse pressure, endothelium function, and arterial stiffness in spontaneously hypertensive rats. Hypertension.

[33] Nyborg N.C., Bevan J.A. (1988). Increased alpha-adrenergic receptor affinity in resistance vessels from hypertensive rats. Hypertension.

[34] de Oliveira A.A., Priviero F., Webb R.C., Nunes K.P. (2022). Increased eHSP70-to-iHSP70 ratio disrupts vascular responses to calcium and activates the TLR4-MD2 complex in type 1 diabetes. Life Sci..

[35] de Oliveira A.A., Nunes K.P. (2020). An additional physiological role for HSP70: Assistance of vascular reactivity. Life Sci..

[36] Touyz R.M., Schiffrin E.L. (1997). Role of calcium influx and intracellular calcium stores in angiotensin II-mediated calcium hyper-responsiveness in smooth muscle from spontaneously hypertensive rats. J. Hypertens..

[37] Nelson L.D., Mashburn N.A., Bell P.D. (1996). Altered sodium-calcium exchange in afferent arterioles of the spontaneously hypertensive rat. Kidney Int..

[38] Taniguchi S., Furukawa K.-I., Sasamura S., Ohizumi Y., Seya K., Motomura S. (2004). Gene expression and functional activity of sodium/calcium exchanger enhanced in vascular smooth muscle cells of spontaneously hypertensive rats. J. Cardiovasc. Pharmacol..

[39] Liu D., Yang D., He H., Chen X., Cao T., Feng X., Ma L., Luo Z., Wang L., Yan Z. (2009). Increased transient receptor potential canonical type 3 channels in vasculature from hypertensive rats. Hypertension.

[40] Wu B., Su C., Wu J., He J., Pan J. (2002). Effects of losartan on intracellular free calcium concentration of aortic smooth muscle cells in spontaneously hypertensive rat. Guangdong Med. J..

[41] Ko Y., Görg A., Appenheimer M., Wieczorek A.J., Düsing R., Vetter H., Sachinidis A. (1992). Losartan inhibits the angiotensin II-induced stimulation of the phosphoinositide signalling system in vascular smooth muscle cells. Eur. J. Pharmacol. Mol. Pharmacol..

[42] d’Uscio L.V., Shaw S., Barton M., Lüscher T.F. (1998). Losartan but not verapamil inhibits angiotensin II-induced tissue endothelin-1 increase: Role of blood pressure and endothelial function. Hypertension.

[43] de Oliveira A.A., Mendoza V.O., Priviero F., Webb R.C., Nunes K.P. (2022). Age-Related Decline in Vascular Responses to Phenylephrine Is Associated with Reduced Levels of HSP70. Biomolecules.

[44] Maeso R., Navarro-Cid J., Muñoz-García R., Rodrigo E., Ruilope L.M., Lahera V., Cachofeiro V. (1996). Losartan reduces phenylephrine constrictor response in aortic rings from spontaneously hypertensive rats. Role of nitric oxide and angiotensin II type 2 receptors. Hypertension.

[45] de PRodrigues S.F., dos Santos R.A., Silva-Antonialli M.M., Scavone C., Nigro D., Carvalho M.H., de Cássia Tostes R., Fortes Z.B. (2006). Differential effect of losartan in female and male spontaneously hypertensive rats. Life Sci..

[46] Bhalla R., Webb R., Singh D., Ashley T., Brock T. (1978). Calcium fluxes, calcium binding, and adenosine cyclic 3′,5′-monophosphate-dependent protein kinase activity in the aorta of spontaneously hypertensive and Kyoto Wistar normotensive rats. Mol. Pharmacol..

[47] Nabika T., Velletri P., Beaven M., Endo J., Lovenberg W. (1985). Vasopressin-induced calcium increases in smooth muscle cells from spontaneously hypertensive rats. Life Sci..

[48] Et-Taouil K., Safar M., Plante G.E. (2003). Mechanisms and consequences of large artery rigidity. Can. J. Physiol. Pharmacol..

[49] Cai Z., Gong Z., Li Z., Li L., Kong W. (2021). Vascular Extracellular Matrix Remodeling and Hypertension. Antioxid. Redox Signal..

[50] Boutouyrie P., Chowienczyk P., Humphrey J.D., Mitchell G.F. (2021). Arterial Stiffness and Cardiovascular Risk in Hypertension. Circ. Res..

[51] Brown I.A.M., Diederich L., Good M.E., DeLalio L.J., Murphy S.A., Cortese-Krott M.M., Hall J.L., Le T.H., Isakson B.E. (2018). Vascular Smooth Muscle Remodeling in Conductive and Resistance Arteries in Hypertension. Arter. Thromb. Vasc. Biol..

[52] Safar M.E., Levy B.I., Struijker-Boudier H. (2003). Current perspectives on arterial stiffness and pulse pressure in hypertension and cardiovascular diseases. Circulation.

[53] Morgan E.E., Casabianca A.B., Khouri S.J., Kalinoski A.L.N. (2014). In vivo assessment of arterial stiffness in the isoflurane anesthetized spontaneously hypertensive rat. Cardiovasc. Ultrasound.

[54] Lindesay G., Ragonnet C., Chimenti S., Villeneuve N., Vayssettes-Courchay C. (2016). Age and hypertension strongly induce aortic stiffening in rats at basal and matched blood pressure levels. Physiol. Rep..

[55] Liu Q., Dong S., Zhou X., Zhao Y., Dong B., Shen J., Yang K., Li L., Zhu D. (2023). Effects of Long-Term Intervention with Losartan, Aspirin and Atorvastatin on Vascular Remodeling in Juvenile Spontaneously Hypertensive Rats. Molecules.

[56] Li J.-S., Sharifi A.M., Schiffrin E.L. (1997). Effect of AT1 angiotensin-receptor blockade on structure and function of small arteries in SHR. J. Cardiovasc. Pharmacol..

[57] Nouri-Majalan N., Nafisi R., Moghadasi-Mousavi S. (2009). Effect of losartan on Doppler sonography indices in kidney transplant patients: A randomized clinical trial. Vasc. Health Risk Manag..

[58] de Oliveira A.A., Priviero F., Delgado A., Dong P., Mendoza V.O., Gu L., Webb R.C., Nunes K.P. (2022). Connecting Aortic Stiffness to Vascular Contraction: Does Sex Matter?. Int. J. Mol. Sci..

[59] Deyl Z., Jelínek J., Macek K., Chaldakov G., Vankov V. (1987). Collagen and elastin synthesis in the aorta of spontaneously hypertensive rats. J. Vasc. Res..

[60] Keeley F.W., Alatawi A. (1991). Response of aortic elastin synthesis and accumulation to developing hypertension and the inhibitory effect of colchicine on this response. Lab. Investig. J. Tech. Methods Pathol..

[61] Keeley F.W., Johnson D.J. (1986). The effect of developing hypertension on the synthesis and accumulation of elastin in the aorta of the rat. Biochem. Cell Biol..

[62] Ito H., Kwan C., Daniel E. (1987). Elastin and elastase-like enzyme change in aorta of rat with malignant hypertension. Exp. Mol. Pathol..

[63] Arribas S.M., Hinek A., González M.C. (2006). Elastic fibres and vascular structure in hypertension. Pharmacol. Ther..

[64] Wang K., Meng X., Guo Z. (2021). Elastin Structure, Synthesis, Regulatory Mechanism and Relationship With Cardiovascular Diseases. Front. Cell Dev. Biol..

