# Supplementary material for: Effect of Antihypertensive Losartan on Ca2+ Mobilization in the Aorta of Middle-Aged Spontaneously Hypertensive Female Rats
Source: J Cardiovasc Dev Dis. 2025 Nov 7;12(11):441. doi: 10.3390/jcdd12110441 (PMC12653655; doi:10.3390/jcdd12110441)
Supplement: Supplementary file 1 [file jcdd-12-00441-s001.zip › jcdd-3934038-supplementary.pdf]

A

Body weight (g)	Groups	mean ± SEM	p- values
	WIS vs. SHR	358.6 ± 6.9	0.0001
	WIS vs WIS+LOS	358 ± 7.0	0.0623
	SHR vs. WIS+LOS	205.6 ± 7.2	0.0001
	SHR vs. SHR+LOS	228.2 ± 2.7	0.0282

B

Mean Arterial Pressure (mm of Hg)	Groups	mean ± SEM	p- values
	WIS vs. SHR	102.5 ± 1.3	0.0001
	WIS vs WIS+LOS	102.3 ± 1.2	0.9983
	SHR vs. WIS+LOS	100.3 ± 3.5	0.0001
	SHR vs. SHR+LOS	107.0 ± 3.1	0.0001

Table S1: Statistical analysis and p-values for the body weight and blood pressure of middle-aged hypertensive females. This table describes the (A) body weight (B) blood pressure of the middle-aged hypertensive females.

A

Area under the curve (AUC)	Groups	mean ± SEM	p- values
	WIS vs. SHR	455.0 ± 38.6	0.0009
	WIS vs WIS+LOS	455.0 ± 37.6	>0.9999
	SHR vs. WIS+LOS	467.1 ± 43.6	0.001
	SHR vs. SHR+LOS	422.5 ± 8.8	0.0003

B

Emax	Groups	mean ± SEM	p- values
	WIS vs. SHR	79.85 ±5.8	0.001
	WIS vs WIS+LOS	79.85 ± 5.7	>0.9999
	SHR vs. WIS+LOS	77.11 ±2.8	<0.0001
	SHR vs. SHR+LOS	71.93 ±4.1	<0.0001

Table S2: Statistical analysis and p-values for Area Under the Curve (AUC) and Emax for the Concentration-Response curve in the aorta of middle-aged hypertensive females. This table describes (A) Area Under the Curve (AUC) and (B) Emax for Concentration-Response Curve in the aorta of middle-aged hypertensive females.

A

	Groups	mean ± SEM	p- values
Ca^{2+} Efflux	WIS vs. SHR	4.7± 0.1	0.0179
	WIS vs WIS+LOS	4.8 ± 0.1	>0.9999
	SHR vs. WIS+LOS	4.5± 0.4	0.0117
	SHR vs. SHR+LOS	4.0± 0.9	0.0033

B

	Groups	mean ± SEM	p- values
Ca^{2+} Influx	WIS vs. SHR	7.8± 0.3	0.0027
	WIS vs WIS+LOS	7.2 ± 0.2	>0.9999
	SHR vs. WIS+LOS	7.8± 0.4	0.0037
	SHR vs. SHR+LOS	7.2 ±0.8	0.0012

Table S3: Statistical analysis and p-values for the Ca^{2+} protocol in the aorta of middle-aged hypertensive females. This table describes the (A) Ca^{2+} efflux and (B) Ca^{2+} influx in the aorta of middle-aged hypertensive females.

A

	Groups	mean ± SEM	p- values
Emax of Phasic (fast)	WIS vs. SHR	39.5 ± 0.7	<0.0001
	WIS vs WIS+LOS	41.6 ± 0.7	>0.9999
	SHR vs. WIS+LOS	41.5 ± 2.5	<0.0001
	SHR vs. SHR+LOS	41.6 ± 1.0	<0.0001

B

	Groups	mean ± SEM	p- values
Emax of Tonic (slow)	WIS vs. SHR	41.4 ± 3.9	<0.0001
	WIS vs WIS+LOS	41.4 ± 4.0	>0.9999
	SHR vs. WIS+LOS	33.9± 3.8	<0.0001
	SHR vs. SHR+LOS	43.2 ±4.8	<0.0001

Table S4: Statistical analysis and p-values for phasic and tonic components of biphasic contraction in the aorta of middle-aged hypertensive females. This table describes (A) phasic and (B) tonic components of biphasic contraction in the aorta of middle-aged hypertensive females.

A

	Groups	mean ± SEM	p- values
Intracellular Ca^{2+} free levels	WIS vs. SHR	1.9 ± 0.1	<0.0001
	WIS vs WIS+LOS	1.9 ± 0.1	0.1088
	SHR vs. WIS+LOS	1.4 ± 0.1	<0.0001
	SHR vs. SHR+LOS	2.4 ±0.1	0.0003

Table S5: Statistical analysis and p-values for intracellular Ca^{2+} levels in the aorta of middle-aged hypertensive females. This table describes the statistical analysis and p-values for the (A) intracellular Ca^{2+} levels in the aorta of middle-aged hypertensive females.

A

	Groups	mean ± SEM	p- values
PWV	WIS vs. SHR	3.0± 0.4	<0.0001
	WIS vs WIS+LOS	2.9 ± 0.4	0.9980
	SHR vs. WIS+LOS	3.3± 0.3	<0.0001
	SHR vs. SHR+LOS	3.2± 0.3	<0.0001

B

	Groups	mean ± SEM	p- values
Diameter	WIS vs. SHR	1.0± 0.04	0.0005
	WIS vs WIS+LOS	1.3 ± 0.04	>0.9999
	SHR vs. WIS+LOS	1.2± 0.01	0.0010
	SHR vs. SHR+LOS	1.0± 0.03	0.6660

C

	Groups	mean ± SEM	p- values
Pulsatility Index (PI)	WIS vs. SHR	1.0± 0.1	0.0001
	WIS vs WIS+LOS	1.0± 0.1	0.9797
	SHR vs. WIS+LOS	1.6± 0.1	0.0004
	SHR vs. SHR+LOS	1.8± 0.02	0.0049

D

	Groups	mean ± SEM	p- values
Resistivity Index (RI)	WIS vs. SHR	0.7± 0.05	0.0004
	WIS vs WIS+LOS	0.7 ± 0.04	0.3388
	SHR vs. WIS+LOS	0.8±0.03	0.0143
	SHR vs. SHR+LOS	0.8±0..02	0.0082

Table S6: Statistical analysis and p-values for PWV, Diameter, Pulsatility Index and Resistivity Index in the aorta of middle-aged hypertensive females. This table describes the statistical analysis and p-values

for the (A) PWV. (B) Diameter. (C) Pulsatility Index. (D) Resistivity Index in the aorta of middle-aged hypertensive females.

A

	Groups	mean ± SEM	p- values
Young's Modulus	WIS vs. SHR	596.3 ± 40	<0.0001
	WIS vs WIS+LOS	596.3 ± 40.9	>0.9999
	SHR vs. WIS+LOS	622.8± 36.9	<0.0001
	SHR vs. SHR+LOS	625.1± 20.4	<0.0001

Table S7: Statistical analysis and p-values for the Young's Modulus in the aorta of the middle-aged hypertensive females. This table describes the statistical analysis and p-values for the (A) Young's Modulus in the aorta of the middle-aged hypertensive females.

A

	Groups	mean ± SEM	p- values
Collagen % of the total vessel area	WIS vs. SHR	19.2 ± 0.6	<0.0001
	WIS vs WIS+LOS	19.1 ± 0.6	>0.9999
	SHR vs. WIS+LOS	19.0 ± 0.6	<0.0001
	SHR vs. SHR+LOS	18.1 ± 0.5	<0.0001

B

	Groups	mean ± SEM	p- values
Collagen % of the media layer	WIS vs. SHR	14.8± 0.8	0.0002
	WIS vs WIS+LOS	14.8 ± 0.8	0.9998
	SHR vs. WIS+LOS	14.9± 0.7	0.0003
	SHR vs. SHR+LOS	14.5 ± 0.5	0.0001

C

	Groups	mean ± SEM	p- values
Elastin %	WIS vs. SHR	9.9± 0.6	0.0006
	WIS vs WIS+LOS	10.0 ± 0.5	>0.9999
	SHR vs. WIS+LOS	9.9 ± 0.4	0.0815
	SHR vs. SHR+LOS	8.3± 0.3	0.0006

Table S8: Statistical analysis and p-values for the Collagen % of the total vessel area, Collagen % of the media layer and Elastin % in the aorta of middle-aged hypertensive females. This table describes the statistical analysis and p-values for the (A) Collagen % of the total vessel area. (B) Collagen % of the media layer. (C) Elastin % in the aorta of middle-aged hypertensive females.
